# Cardiac angiosarcoma: a case report of a young female with pulmonary metastasis

**DOI:** 10.1186/s43044-022-00277-7

**Published:** 2022-05-21

**Authors:** Mehran Pournazari, Shirin Assar, Dena Mohamadzadeh, Maryam Mahdian, Setareh Soltani

**Affiliations:** 1 Rheumatology Department, Clinical Research Development Center, Imam Reza Hospital, Kermanshah University of Medical Sciences, Kermanshah, Iran; 2 Student Research Committee, Kermanshah University of Medical Sciences, Kermanshah, Iran; 3 Clinical Research Development Center, Taleghani and Imam Ali Hospital, Kermanshah University of Medical Sciences, Kermanshah, Iran

**Keywords:** Angiosarcoma, Cardiac angiosarcoma, Pulmonary metastasis

## Abstract

**Background:**

Angiosarcoma is a malignant rare tumor that originates from vascular endothelial cells that cover lymphatic or blood vessels. Cardiac angiosarcoma is the most prevalent sarcoma entail the heart. It has low incidence rate and poor prognosis. Our effort through this report was raising awareness of uncommon manifestations of this disease and showing the importance of appropriate diagnosis and treatment.

**Case presentation:**

We present a case of cardiac angiosarcoma in a young female whose symptoms included dyspnea and hemoptysis with a history of pericardial effusion and a past history of cardiac surgery for suspected atrial Myxoma. She had history of several hospitalizations and relapse of symptoms a few months after each hospital discharge.

**Conclusions:**

The unspecific symptoms of cardiac angiosarcoma made it difficult to make in time diagnose and appropriate treatment. Awareness of unspecific presentations of cardiac angiosarcoma is necessary for proper diagnosis and treatment while delayed diagnosis may worsen the prognosis and even lead to death.

## Background

Only 20% of cardiac tumors are malignant [1] and nearly 75% of primary cardiac tumors are benign. Cardiac angiosarcomas are scarce sarcomas of soft tissue and have a poor prognosis [2].

In a review of 45 cases of cardiac angiosarcomas, 41 of them (93%) were found in the right atrium. Nevertheless, the sinusoidal pattern seems to be pathognomonic for angiosarcomas but they can have the appearance of benign tumors. Thus, histology evaluation is difficult [3]. The prevalence of primary cardiac angiosarcomas is higher in male in a two-thirds ratio [4]. As a standardized management, complete or partial surgery resection is still the best palliative option. However, chemotherapy and target therapy are so confined [2, 5].

The reports of patients with familial history of primary cardiac angiosarcoma determine 4 months as a mean survival time [6].

This case report describes a case of cardiac angiosarcoma that had complications during 3 years before definite diagnosis. In spite of being under observation and having a cardiac surgery due to a suspected myxoma in these years, it could not be diagnosed until the last biopsy that was taken in our department.

## Case presentation

The patient was a 20-year-old Iranian female who was a dentistry student in Moscow, Russia. She presented with dyspnea, hemoptysis and pain in the upper extremities since few days prior to the recent hospitalization and she was admitted to the intensive-care unit (ICU) of Kermanshah Hospital, Iran on May 4th, 2019. She denied fever or chest pain. There was no history of alcohol, tobacco or illicit drug use. She claimed that she had been hospitalized for three times between 2017 and 2019. The first hospitalization occurred on April, 2017 when she was in Isfahan City, Iran for holidays. Her chief complaints were palpitation, sudden dyspnea and chest pain. She was hospitalized in cardiac care unit (CCU) for few weeks. Electrocardiography (ECG) was taken and sinus tachycardia was the only finding. Laboratory results were unremarkable except for elevated cardiac enzymes. Her symptoms disappeared temporarily by taking Metoprolol 50 mg daily. One year later in early 2018, she developed dyspnea. Pericardial effusion was detected in echocardiography and she was treated with colchicine 1 mg and non-steroidal anti-inflammatory drugs (NSAIDs). She was hospitalized for the third time in late 2018, in Tehran City, Iran. Her symptoms included dyspnea and hemoptysis. Pulmonary nodules were found in chest computerized tomography (CT) scan and a mass suspected to be a Myxoma was found in the right atrium in echocardiography. She underwent an open cardiac surgery, and the pathologic report of the cardiac mass was clot. The report of the CT guided biopsy of pulmonary nodules didn’t confirm malignancy. The differential diagnosis considered for pulmonary nodules were Tuberculosis (TB), malignancy and vasculitis but there was no document for any of them. Therefore, prednisolone 50 mg daily was started for any unknown inflammation supposed to exist. She was on prednisolone 25 mg daily when presented to our hospital.

On the general examination she had tachycardia. Her vital signs were: Temperature = 37, Respiratory Rate = 16, Pulse Rate = 110, Blood Pressure = 110/70. Fine crackles were found on the respiratory system examination. Examination of the heart and abdomen were within normal limits.

Laboratory tests on the first day of admission showed, leukocytosis with white blood cells (WBC) = 19.5 × 10^3^/mm^3^ (normal: 4–10 × 10^3^/mm^3^), red blood cell (RBC) 4.11 × 10^6^/mm^3^ (normal: 4.2–5.4 × 10^6^/mm^3^), anemia (hemoglobin = 9.9 mg/dl) (normal: 12–16 mg/dl), ferritin 268 ng/ml (normal: 10–124 ng/ml), reticulocyte count 9%, platelet count 267 × 10^3^/mm^3^ (normal: 140–440 × 10^3^/mm^3^), partial thromboplastin time (PTT) 37 s (normal: 24–35 s), international normalized ratio (INR) 1.2 (normal = 1), creatinine 0.7 mg/dl (normal: 0.5–1.3 mg/dl), lactate dehydrogenase (LDH) 1157 IU/ml (normal: 225–500 IU/ml), bilirubin total 1.8 mg/dl (normal: 0.2–1.4 mg/dl), bilirubin direct 0.5 mg/dl (normal: 0–0.4 mg/dl), erythrocyte sedimentation rate (ESR) 3 mm/hr, and C-reactive protein (CRP) was negative. Liver function tests and urinalysis (U/A) were normal. Urine and blood cultures were sterile. Arterial blood gas (ABG) analysis showed PH = 7.47, Pco2 = 32 mm Hg, Po2 = 32 mm Hg. Additional laboratory investigations were done. Tumor marker CA-125 was 47.7 U/ml (normal > 46 U/ml) and CA19-9 was 5.19 U/ml (normal > 37 U/ml). Viral tests for HIV, Hepatitis A, B, and C were negative. Immunological workup was unremarkable. Antinuclear antibody, anti-ds DNA antibody, perinuclear ANCA (P-ANCA), Anti RO, Anti La, cytoplasmic ANCA (C-ANCA), angiotensin converting enzyme (ACE), C3, C4, CH50, APS (anti-phospholipid antibody), Anti CCP (cyclic citrullinated peptide), RF (rheumatoid factor) and anti-GBM (glomerular basement membrane) antibody were negative. Laboratory workup for TB and fungal infections revealed negative results.

**Fig. 1:**
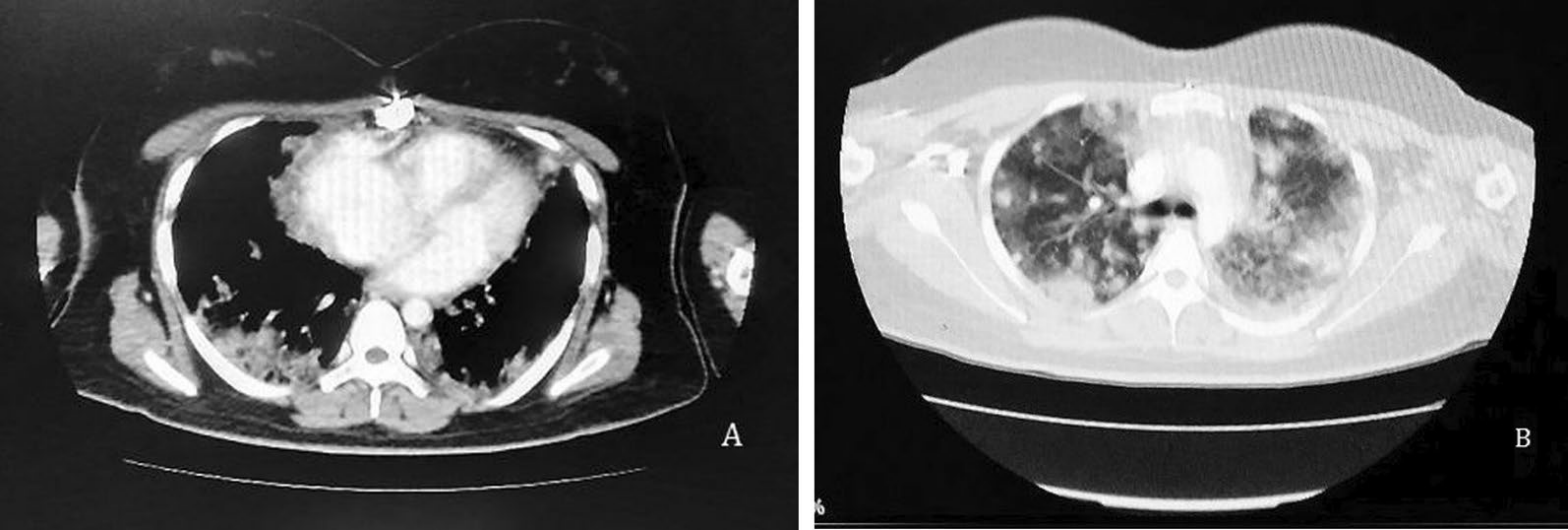
High-resolution computed tomography (HRCT) of the patient showing **A** mild pleural effusion with dilated left atrium and **B** bilateral pulmonary multi nodular consolidations

Chest X-ray presented the blunting of the left costophrenic angle without cardiomegaly. Echocardiography reported mild mitral regurgitation (MR), tricuspid regurgitation (TR), pulmonary artery pressure (PAP) of 35 mm Hg without pleural effusion. A computed tomography (CT) scan of the abdomen and pelvis was normal. Report of chest high-resolution computed tomography (HRCT) without contrast demonstrated a mild pleural effusion in left side, bilateral pulmonary multi nodular consolidations (maximum size: 16 mm), with air bronchogram in inferoposterior area of both lungs that were suspicious for metastasis, left atrium was dilated with rough wall and two filling defect of 15 × 22 mm (Fig. 1). Breast and thyroid sonography were unremarkable.

**Figure 2:**
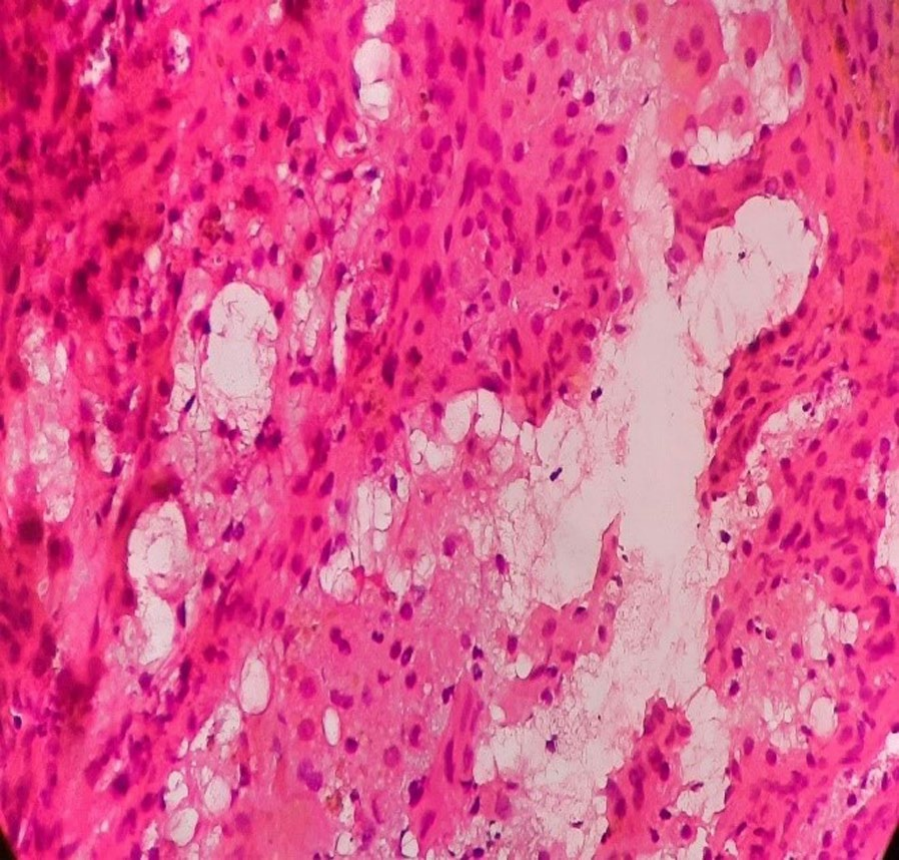
Left lung biopsy specimen showing well-formed vasoformative channels suggesting cardiac angiosarcoma (Hematoxylin and eosin stain, 40 ×)

She underwent bronchoscopy. The report of the sample of bronchoalveolar lavage showed macrophage cells and polymorphonuclear leukocytes (PMN) with bloody discharge. The first biopsy of the left lung could not detect any evidence in favor of malignancy because its specimen hadn’t enough volume. Result of the second biopsy of left lung tissue measuring: 6 × 3 × 1.5 cm was suggestive for epithelial tumor without necrosis showing high mitosis rate and many vascular channels indicative for metastatic angiosarcoma (Fig. 2).

The patient developed hypoxia needing mechanical ventilation. Unfortunately, she succumbed to illness on the 33^rd^ day of hospitalization. Eventually the report of the second left lung biopsy revealed Spindle to epithelioid cells with mild atypia accompanied with small vascular channels with scattered mitotic figures and angiosarcoma was confirmed.

## Discussion

Cardiac angiosarcoma is so scarce despite being the majority of primary malignant cardiac tumors [3, 7]. Due to the variability of presentations that most depends on its location, diagnosis is so difficult [7, 8]. Other reason for difficulty in diagnosis is the unspecific presentations such as dyspnea, chest pain, cough, also hemoptysis and syncope [8]. The disease is more common in young males between 3rd to 5th decades of life [4]. It has a very poor prognosis and its mean survival is between 9.6 to 16.5 months [9]. The location of nearly 90% of cardiac angiosarcomas is in the right atrium with high property of pericardial involvement [3, 8].

The imaging examinations like trans-esophageal echocardiography (TEE) can display its location, extension and also the type of the tumor [3, 8]. The pseudo negative results are also reported because of being small in size, or locating in the posterior surface of myocardium [8]. Computed tomography (CT) and magnetic resonance imaging (MRI) are useful to present the invasion and metastasis and the other information such as tumor size and localization [9]. In our case, the report of HRCT showed pulmonary metastasis and dilatation and rough wall of left atrium with filing defects in it, and its decisive diagnosis was proved by the left lung biopsy.

Surgery is still the best choice for treatment and for its early metastasis neo-adjuvant chemotherapy is recommended. In Blackmon SH research for right heart sarcomas, mean survival time of this combination therapy was 27 months and the longest survival was 9.5 years [10]. In the left sided sarcoma cardiac transplantation can be performed. Using the combination of neo-adjuvant chemotherapy and ifosfamide (IFO) before resection surgery can result in mean survival time rising to 15.5 months in right sided and to 20 months in left [11]. One research showed the increase of median survival to 4.8 months and total survival to 9.9 months with locating anthracycline in the first line of chemotherapy [12]. The approbated treatment is complete resection surgery and neo-adjuvant chemotherapy [13]. Nevertheless, total resection was not possible in our case because of being a super invasion and having distal metastasis and delayed diagnosis which led to losing the chance of having successful operation [14]. The prognosis of these sarcomas is so poor. Infiltration of myocardium, cardiac tamponade, obstruction of blood flow and remote metastasis are the causes of unpreventable death in our case [15].

The differential diagnoses of the angiosarcomas include primarily benign cardiac tumors intracardiac, vegetations and intracardiac thrombi [16]. Our patient underwent a cardiac surgery one year prior to present hospitalization due to suspected cardiac myxoma and the pathologic examination of the tissue specimen was consistent with thrombus.

This case was a predicament because of the inability to access the report of the diagnostic examinations that were done by different departments of other cities during the past 3 years, despite our effort to connect them.

## Conclusions

Angiosarcoma is a malignant and rare tumor which may present with various clinical scenarios. Awareness of unspecific presentations of cardiac angiosarcoma is necessary for proper diagnosis and treatment. Delayed diagnosis may worsen the prognosis and even lead to death.

## Data Availability

The data are available for sharing.

## Abbreviations

ICU: Intensive-care unit
CCU: Cardiac-care unit
ECG: Electrocardiography
CT: Computerized tomography
NSAID: Non-steroidal anti-inflammatory drugs
TB: Tuberculosis
TEE: Trans-esophageal echocardiography
MRI: Magnetic resonance imaging
MR: Mitral regurgitation
TR: Tricuspid regurgitation
HRCT: High-resolution computed tomography
IFO: Ifosfamide
